# Spatial and temporal variations of particulate organic carbon in the Yellow-Bohai Sea over 2002–2016

**DOI:** 10.1038/s41598-018-26373-w

**Published:** 2018-05-22

**Authors:** Hang Fan, Xiujun Wang, Haibo Zhang, Zhitong Yu

**Affiliations:** 1 College of Global Change and Earth System Science, Beijing Normal University, Beijing, China; 2 Key Laboratory of Soil Contamination Bioremediation of Zhejiang Province, Zhejiang A&F University, Hangzhou, China

## Abstract

The Yellow-Bohai Sea (YBS) is a typical marginal sea in the Northwest Pacific Ocean; however, little is known about the dynamics of particulate organic carbon (POC) and underlying mechanisms. Here, we analyze the spatial and temporal variations of surface POC derived from MODIS-Aqua during 2002–2016. Overall, POC is higher in the Bohai Sea (315–588 mg m^−3^) than in the Yellow Sea (181–492 mg m^−3^), and higher in the nearshore than in the offshore. Surface POC is highest in spring in the YBS, and lowest in winter (summer) in the Bohai Sea (the Yellow Sea). The spatial and seasonal patterns of POC are due to combined influences of primary productivity, water exchange, sediment resuspension and terrestrial inputs. Surface POC shows an overall decreasing trend prior to 2012 followed by an upward trend until 2015 in the YBS, which is almost opposite to chlorophyll; the decrease (increase) may result from strengthened (weakened) water exchange with the East China Sea through the Yellow Sea Warm Current. Declined terrestrial runoff is also partly responsible for the decrease prior to 2012. Our study suggests that water exchange and sediment resuspension are dominant factors regulating the spatial and temporal variability of POC in the YBS.

## Introduction

The concentration of carbon dioxide (CO_2_) in the atmosphere has increased from approximately 280 parts per million in the beginning of the Industrial Era to about 400 ppm in 2013^1^. Elevated levels of CO_2_ in the atmosphere can lead to problems such as global warming and ocean acidification^2^. The ocean is a major sink of atmospheric CO_2_ and plays a large role in mitigating the global warming^1,3^. The marginal seas account for only about 7% of the total ocean surface area, but contribute 14–30% of the oceanic primary productivity^4^. Therefore, the marginal seas may play an important role in the oceanic carbon cycle.

Biological productivity is significantly higher in the marginal seas relative to the open oceans, due to higher rates of nutrient supply through upwelling, riverine inputs, and/or terrestrial runof f ^5^. As a result, accumulation rate of organic carbon in the marginal seas is 8–30 times (per unit area) higher than that in the open oceans^5^. Particulate organic carbon (POC) is an important part of the organic carbon in the ocean, which has a direct linkage to the biological pump. There have been some studies on POC dynamics in the marginal seas. For example, a recent study in the northern Barents Sea showed the maximum POC at 20–40 m^6^. Hung, *et al*.^7^ reported that 27–93% of the POC flux in the East China Sea might be from resuspension of bottom sediments.

The Yellow-Bohai Sea is a marginal sea in the Northwest Pacific Ocean. There have been field studies of the carbon cycle in the Yellow-Bohai Sea. Previous studies have demonstrated that the Yellow-Bohai Sea is generally a sink of the atmospheric CO_2_, but there are some sections acting as a CO_2_ source^8^. Limited studies have shown that the Yellow-Bohai Sea is a significant repository of sedimentary total organic carbon with large spatial variability^9,10^; there is a significant seasonal and spatial variability in the POC of the Yellow-Bohai Sea, which may be associated with anthropogenic perturbations and oceanic physical processes^10–13^.

The main anthropogenic impacts in the Yellow-Bohai Sea may include terrestrial runoff and river discharge of nutrients and/or organic matters that promote biological activity^14,15^ and lead to higher levels of POC nearshore. On the other hand, oceanic physical processes can affect the biological and biogeochemical processes with implications for the temporal and spatial variability of POC. For example, the local movement of Yellow Sea Cold Water Mass can block the upward transportation of high-nutrient water and suspended particulate matters in the centers of north Yellow Sea and south Yellow Sea, which is often pronounced in the summer season^11,16,17^. In addition, physical and biogeochemical processes in the Yellow-Bohai Sea are largely influenced by the current systems that are linked with the northwest Pacific Ocean through the East China Sea, which may be associated with large-scale climate modes. In particular, the Yellow Sea Warm Current, as a branch of the Kuroshio Current extension, may be affected by the El Niño-Southern Oscillation (ENSO) and Pacific Decadal Oscillation (PDO)^18–20^.

Apart from the ENSO and PDO, the North Pacific Gyre Oscillation (NPGO) can also affect the physical and biogeochemical processes at interannual or decadal time scales in the marginal seas of the northwest Pacific^21^. A recent study shows that the temporal variability of sea surface height in the China sea is strongly modulated by the ENSO at the interannual time scale, but largely influenced by the NPGO and PDO at the decadal time scales^22^. Similarly, these major climate modes may also have influences on the physical and/or biogeochemical processes of the Yellow-Bohai Sea at interannual to decadal time scales. However, field measurements are too sparse in the Yellow-Bohai Sea to warrant the analyses of temporal variation of POC at interannual and longer time scales. On the other hand, ocean color remote sensing can provide large-scale, continuous and dense ocean data^23^. There have been studies of the spatial and temporal variations of POC at large scales by using remote sensed POC data^24–26^. For example, Stramska and Bialogrodzka^24^ applied ocean color data derived from SeaWiFS and MODIS to explore the seasonal and interannual change of POC in the Barents Sea.

There has been limited analysis of POC in the Yellow-Bohai Sea using remote sensed data (i.e., Cong, *et al*.^27^); little is done to evaluate how POC changes over the seasonal to interannual time scales in this region. Here, we use high-resolution POC data derived from MODIS-Aqua from July 2002 to December 2016 to study the spatial and temporal variability of surface POC in the Yellow-Bohai Sea. To explore the influence of large-scale climate modes on the dynamics of surface POC, we examine the correlations of surface POC with PDO, NPGO and ENSO indices. The objective of this study is to improve the understanding of the impact of human activities and natural processes on the spatial and temporal variations of POC in the Yellow-Bohai Sea.

## Regional Setting

The Yellow-Bohai Sea is a semi-closed marginal sea located between China mainland and the Korean Peninsula, which is also a shallow sea with an average depth of 31 m, and the deepest (140 m) found in the Yellow Sea^28^. There are more than 17 rivers flowing into the Bohai Sea, including the Yellow River (the second largest sediment-load river in the world), Liao River, Luan River and Haihe River, which deliver a large amount of industrial and agricultural wastewater and sediment^29–31^.

The Yellow Sea has currents and water mass with remarkable seasonal variations, i.e. the Yellow Sea Cold Water Mass and the Yellow Sea Warm Current. The Yellow Sea Cold Water Mass is a bottom pool of remnant Yellow Sea Winter Water^16^, which is most significant in summer^28,32^. The existence of the Yellow Sea Cold Water Mass causes a strong stratification in the centers of north Yellow Sea and south Yellow Sea^16,33^. The Yellow Sea Warm Current is the main component of the Yellow Sea circulation, which initiates in fall, becomes strongest in winter then weaker in spring and weakest in summer^34^. The Yellow Sea Warm Current, as a branch of the extension of the Kuroshio Current (a western boundary current of the North Pacific Ocean), is the main source of outside seawater for the Yellow-Bohai Sea and the water exchange channel between the Yellow Sea and the East China Sea^35^. The existing of the Yellow Sea Cold Water Mass and changes in the Yellow Sea Warm Current would have large impacts on physical and biogeochemical processes in the Yellow-Bohai Sea at seasonal-to-interannual time scales.

## Results and Discussions

### Comparison of remote sensed POC with *in situ* data

In order to verify the reliability of remote sensed POC for the Yellow-Bohai Sea, we collected *in situ* POC data from published articles, and compared with the remote sensed POC over the same period and area. As shown in Table 1, POC levels are similar between the field measurements (190–459 mg m^−3^) and the remote sensed estimates (259–487 mg m^−3^); the relative differences are small (2–25%) except from in the Yellow Sea in 2012 (49%). However, the highest and lowest values are found during different periods and/or in different regions. For example, the highest POC is found during Apr-May 2010 from the field measurement, but in May 2012 from remoted sensed estimate in the Bohai Sea; the lowest POC is found in Nov 2012 from the field measurement but during Jun-Jul 2013 from remoted sensed estimate in the Yellow Sea. Nevertheless, both field measurement and remote sensed estimate show the highest values in spring.

**Table 1:** Comparison of *in situ* and remote sensed POC (mg m^−3^) over the same period and area.

Purview	Area	*In situ*	Remote sensed	Relative difference (%)^d^
		Mean (S.D.)		
Apr-May 2010	118°E–121°E, 37.5°N–40°N (Bohai Sea)	459 (151)^a^	422 (54.4)	−8
Apr-May 2010	120°E–124°E, 32°N–38.5°N (Yellow Sea)	325 (304)^a^	333 (124)	2
Sep 2010	118°E–121°E, 37.5°N–40°N (Bohai Sea)	432 (241)^a^	419 (71.9)	−3
Sep 2010	120°E–124°E, 32°N–38.5°N (Yellow Sea)	285 (221)^a^	266 (116)	−7
May 2012	118°E–121°E, 37.8°N-40°N (Bohai Sea)	420 (170)^b^	487 (94)	16
May 2012	121°E–124°E, 32°N–39°N (Yellow Sea)	200 (180)^b^	297 (155)	49
Nov 2012	118°E–121°E, 37.8°N–40°N (Bohai Sea)	290 (140)^b^	339 (31.4)	17
Nov 2012	121°E–124°E, 32°N–39°N (Yellow Sea)	190 (140)^b^	283 (98.7)	49
Jun-Jul 2013	118.7°E–121°E, 37.5°N–40°N (Bohai Sea)	441 (111)^c^	383 (79.1)	−13
Jun-Jul 2013	121°E–124°E, 37.5°N–39°N (North Yellow Sea)	347 (131)^c^	259 (98.2)	−25
Jun-Jul 2013	121°E–124°E, 31°N–37.2°N (South Yellow Sea)	316 (277)^c^	269 (113)	−15

^a^Shang^61^; ^b^Liu, *et al*.^10^; ^c^Zhang, *et al*.^11^.

^d^Relative difference is calculated as: (estimation value − measured value)/measured value * 100%.

### Spatial pattern

**Figure 1:**
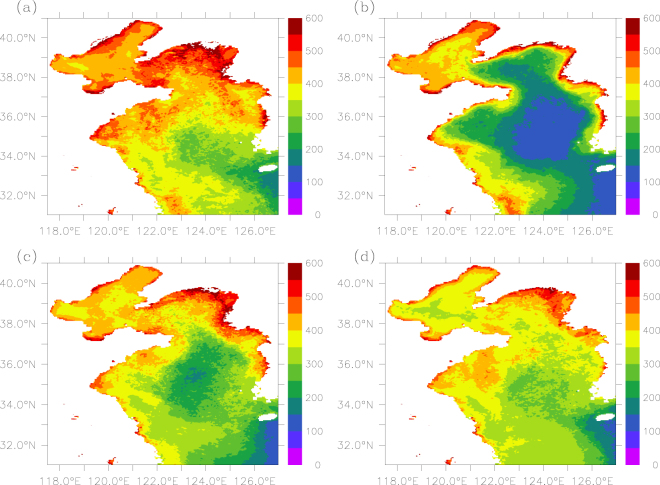
Spatial distribution of POC (mg m^−3^) in (**a**) spring, (**b**) summer, (**c**) fall, and (**d**) winter over 2003–2016. The figure was generated by using Ferret v7.3 (http://ferret.pmel.noaa.gov/Ferret/).

Figure 1 presents the spatial distribution of remote sensed POC in the Yellow-Bohai Sea for the four seasons. Apparently, POC concentration is higher in the Bohai Sea and north Yellow Sea than in the south Yellow Sea, with an average of 413 ± 44, 369 ± 53, 316 ± 67 mg m^−3^, respectively (Table 2). In general, there is a decreasing trend from the nearshore to offshore in all four seasons.

**Table 2:** POC (mg m^−3^) values of different seasons in the three sub-regions.

Season	Bohai Sea		North Yellow Sea		South Yellow Sea	
	Mean $\pm$ S.D.	Anomaly	Mean $\pm$ S.D.	Anomaly	Mean $\pm$ S.D.	Anomaly
Spring	452 $\pm$ 53.6	9	459 $\pm$ 47	90	373 $\pm$ 56.1	57
Summer	415 $\pm$ 52.3	2	245 $\pm$ 84.8	−124	240 $\pm$ 104	−76
Fall	411 $\pm$ 36.2	−2	391 $\pm$ 53.1	22	308 $\pm$ 65.4	−8
Winter	374 $\pm$ 35	−39	380 $\pm$ 28.7	11	342 $\pm$ 40.8	26

There is evidence that primary productivity has large impacts on the spatial distribution of POC in some marginal seas, e.g. the East China Sea and the Barents Sea^6,36^. An early study indicates that primary production is higher in the Bohai Sea than in the Yellow Sea^37^. As an almost closed marginal sea, the Bohai Sea would receive a large amount of nutrients by terrestrial inputs, which can stimulate biological activities and lead to higher rates of primary production^31^. A field study shows a large spatial variability in nutrients in the Yellow-Bohai Sea, with a downward trend from the nearshore to the offshore^15^, which implies higher rates of biological production thus higher levels of POC in the water column near shore.

Apart from the local production, other processes would also affect the spatial distribution of POC. Many studies have showed that rivers often deliver a large amount of organic carbon annually to the coastal oceans^38–40^. For example, Trefry, *et al*.^41^ reports that the Mississippi River (in the USA) transports a large amount of POC into the Gulf of Mexico, which decreases sharply within a few kilometers from the river mouth. A field study conducted in spring and fall seasons (i.e., May and November, 2012) in the Yellow-Bohai Sea shows that the magnitude and variability of organic carbon is largely influenced by primary productivity, followed by sediment resuspension, riverine input and water exchange with the East China Sea^10^. In general, POC content is high in shallow waters because of resuspension of sedimentary organic matters, thus one may expect that the Bohai Sea (and the nearshore waters), with shallow waters, would have higher levels of POC in the water column. In addition, more terrestrial runoff into the Bohai Sea (relative to the Yellow Sea) would also deliver extra organic carbon into the basin^42,43^. On the other hand, water exchange between the Yellow Sea and the East China Sea would lead to reduced POC concentration in the Yellow Sea because POC content in the Yellow Sea is approximately twice of that in the East China Sea^44^.

### Seasonal pattern

**Figure 2:**
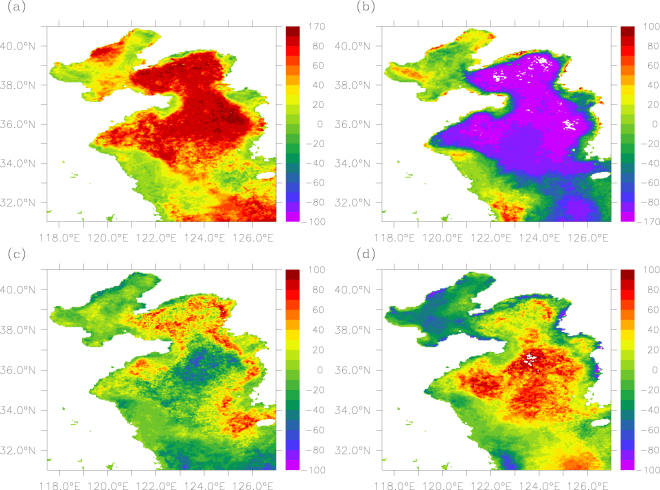
Spatial distribution of the difference between the season mean and annual mean POC (mg m^−3^) for (**a**) spring, (**b**) summer, (**c**) fall, and (**d**) winter. The figure was generated by using Ferret v7.3 (http://ferret.pmel.noaa.gov/Ferret/).

Our analyses indicate that surface POC in the Bohai Sea is highest in spring and lowest in winter, with mean values of 452, 415, 411 and 374 mg m^−3^ in spring, summer, fall and winter, respectively (Table 2). However, an earlier field study shows that the primary productivity in the Bohai Sea is higher in summer than in spring^45^, implying that there may be either extra POC inputs (e.g., via riverine input or resuspension) in spring or reduction of POC in summer (due to removal process or dilution). Given that the POC flux from the Yellow River into the Bohai Sea in summer accounts for about 86% of the total annual input^39^, one may rule out the first possibility, i.e., higher rates of POC inputs in spring through riverine input. There is evidence that the suspended sediment in the surface of the Bohai Sea is higher in spring than in summer^46^. As demonstrated in Fig. 2, the decrease of POC from spring to summer occurs mainly in the central part that connects with the north Yellow Sea. There is evidence of significant water exchange between the Bohai Sea and the Yellow Sea in summer, i.e., “the outflow in the upper layer and the inflow in the bottom layer of the Bohai Strait”^47^, which would cause a reduction of summer POC in the Bohai Sea (i.e., the dilution effect).

For the Yellow Sea, surface POC is highest in spring and lowest in summer except in the coastal area of the south Yellow Sea (Fig. 2). A very earlier study showed that the primary productivity was higher in summer than in winter during 1984–1985^48^; a recent study of the north Yellow Sea revealed that the primary productivity in the water column was about three times higher in summer (mid-July to early August) than in winter (early January). However, our study shows that POC in winter is higher than in summer, implying that there may be processes causing a decrease in summer POC and/or an increase in winter POC. According to Table 2 and Fig. 1b, the spatial variability of POC is largest (104 mg m^−3^) in summer with extremely low values (~100 mg m^−3^) found in the center of the south Yellow Sea. The extremely low POC values in summer may be linked with the Yellow Sea Cold Water Mass that can causes a strong stratification (thus weak vertical mixing) and less upward supply of suspended POC^17^. On the other hand, there is evidence of resuspension enhancing from fall to winter^49^, which leads to an increasing trend in POC over the second half of the year. There is also evidence of pronounced horizontal transportation of suspended particulate matters in winter in the Yellow Sea^49^, which may be responsible for the weak spatial variability in winter POC (see Fig. 1d).

### Interannual variability

**Figure 3:**
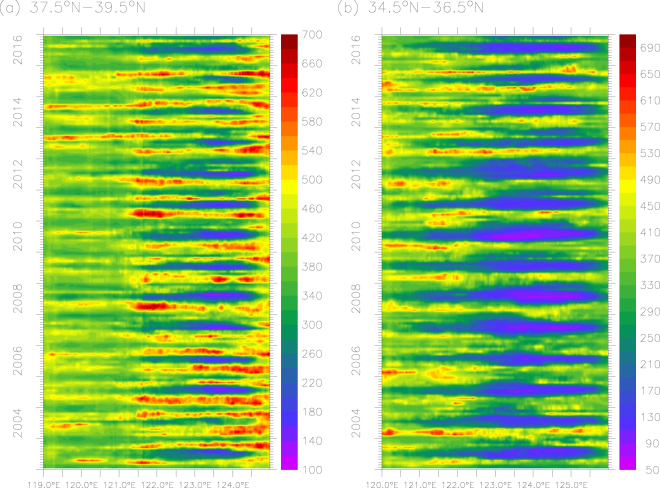
The interannual variability of POC (mg m^−3^) over two-degree band for (**a**) the Bohai Sea-north Yellow Sea (37.5°N–39.5°N) and (**b**) south Yellow Sea (34.5°N–36.5°N). The figure was generated by using Ferret v7.3 (http://ferret.pmel.noaa.gov/Ferret/).

**Figure 4:**
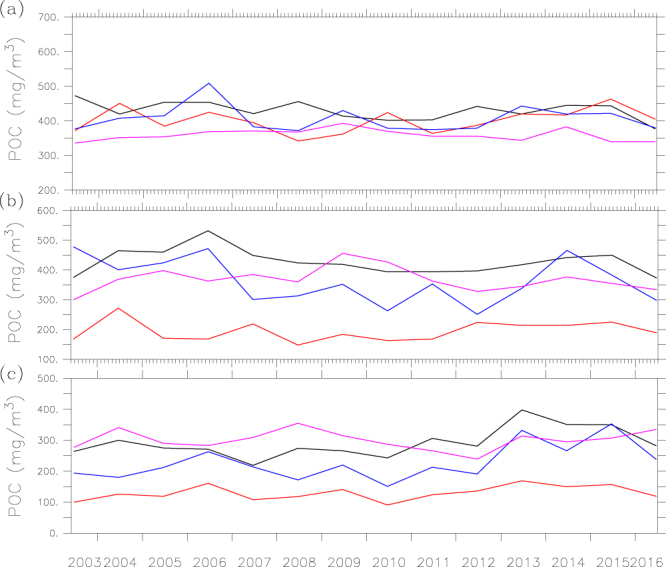
Mean POC (mg m^−3^) in the representative boxes of (**a**) the Bohai Sea (119.5°E–120.5°E, 38°N–39°N), (**b**) the north Yellow Sea (123°E–124°E, 37.5°N–38.5°N), (**c**) the south Yellow Sea (123°E–124°E, 34°N–35°N) in spring (black lines), summer (red lines), fall (blue lines) and winter (purple lines). The figure was generated by using Ferret v7.3 (http://ferret.pmel.noaa.gov/Ferret/).

We evaluate the temporal variability of POC over two bands: 37.5°N–39.5°N and 34.5°N–36.5°N, in which the north band covers both the Bohai Sea and north Yellow Sea. Figure 3a shows a strong seasonal to interannual variability of POC in the Bohai Sea (119°E–121°E), with anomalously high values found during the fall in 2013 and 2014. There are also strong seasonal and interannual variations of POC in the Yellow Sea especially in the south Yellow Sea (Fig. 3a,b). To further assess the interannual variability, we examine the time series of mean POC for each season in the central sections of the Bohai, north and south Yellow Sea. Overall, interannual variability is strongest in fall in all three sub-regions (Fig. 4). Interestingly, interannual variability is weakest in spring in the central Bohai Sea, but in summer in the centers of both north and south Yellow Sea. Surface POC in the central north Yellow Sea has a distinct decrease of POC in fall prior to 2012 and an increase during 2012–2015. Surface POC shows a significant increasing trend in the south Yellow Sea in spring and fall during 2012–2015.

**Figure 5:**
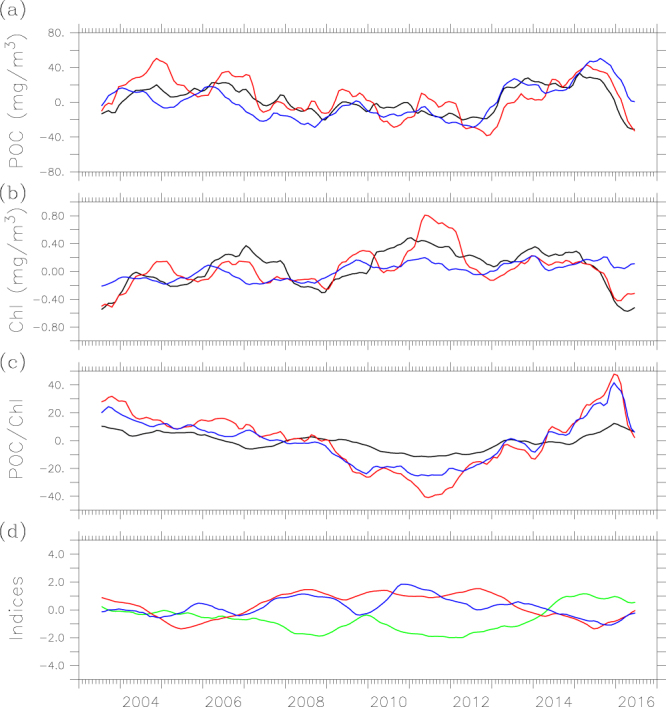
The interannual variability of (**a**) POC, (**b**) chlorophyll, and (**c**) POC chlorophyll ratio in the Bohai Sea (black line), north Yellow Sea (red line) and south Yellow Sea (blue line), and (**d**) time series of PDO (green line), NPGO (purple line) and SOI (blue line) indices. The figure was generated by using Ferret v7.3 (http://ferret.pmel.noaa.gov/Ferret/).

To evaluate the mechanisms responsible for the interannual variability of POC in the Yellow-Bohai Sea, we first analyze the anomaly of satellite derived chlorophyll and temporal variability in nutrients. Figure 5b shows that there is an overall increasing trend in chlorophyll prior to year 2012, indicating an enhancement in biological activity over this period. Indeed, there is evidence that the amount of nutrients of the Yellow-Bohai Sea had an increasing trend from 2002 to 2011^50–52^. However, POC shows a general decreasing trend prior to 2012 (Fig. 5a), implying that the relatively higher POC content in the early 2000s may be attributable to more allochthonous sources (e.g., through riverine inputs). Based on the analyses of POC fluxes from the major rivers to the Yellow-Bohai Sea, there was indeed a decreasing trend during the period of 2003–2009^42^.

There is a distinct increasing trend in surface POC (mainly in fall), but chlorophyll shows little change from 2012 to mid-2015, implying non-biological processes responsible for the increased POC in the Yellow-Bohai Sea, which may include riverine inputs, resuspension of sediment and water exchange with the East China Sea^10^. According to the data from the Bulletin of China River Sediment (http://www.mwr.gov.cn/sj/tjgb/zghlnsgb/), the sediment load into the Yellow River decreased significantly post 2013, implying that riverine inputs might not be responsible for the elevated POC. On the other hand, the recent study of Yin, *et al*.^53^ indicates that the interannual variability of suspended sediment is influenced by the Yellow Sea Warm Current that flows from the East China Sea to the Yellow Sea, and transports the waters of the Kuroshio Current^54^. The Yellow Sea Warm Current was strong during the winter of 2012/2013^55^, but weak during the winter of 2014/2015 owing to the weak East Asian winter monsoon^56,57^, which implies that the water exchange with the East China Sea was weak post 2013. Because POC concentration was higher in the Yellow Sea than in the East China Sea^10^, water exchange between the Yellow-Bohai Sea and East China Sea would result in lower POC in the Yellow Sea (the dilution effect); the weaker Yellow Sea Warm Current would have a less dilution effect on POC in the Yellow-Bohai Sea, which might be partly responsible for the increased POC. Interestingly, surface POC was also anomalously high during 2012–2015 in the northwest Pacific Ocean^58^, which could have influence on the POC dynamics of the Yellow-Bohai Sea through the current system.

### Climate variability influences on POC

There is evidence that the presence of El Niño (La Niña) could weaken (strengthen) the East Asian winter monsoon^59^. The study of Li^56^ reveals that a weak (strong) East Asian winter monsoon can lead to a weak (strong) Yellow Sea Warm Current, resulting in a weak (strong) water exchange between the Yellow-Bohai Sea and the East China Sea. There were several La Niña events during 2002–2012, i.e., 2005–2006, 2007–2009 and 2010–2011, with continuous strong winter monsoon for six years until the winter 2012/2013^55^, which implies that the Yellow Sea Warm Current might be strong during 2005–2011. On the other hand, there were also weak El Niño events during the period of our study, i.e. the winter of 2012/2013 and 2013/2014, and a strong El Niño event during 2014–2015, resulting in a weak winter monsoon. Apart from ENSO, PDO can also affect the East Asia winter monsoon, i.e., when PDO is in a warm phase, the winter monsoon is weaken^57^. Apparently, ENSO and PDO might have impacts on the dynamics of surface POC in the Yellow-Bohai Sea through the influence on the East Asian winter monsoon. In addition, a study shows that the NPGO can also affect the physical and biogeochemical processes at interannual or decadal time scales in the marginal seas of the northwest Pacific^21^.

To explore the influence of climate modes on the POC in the Yellow-Bohai Sea, we assess the relationship of surface POC with the three climate modes (i.e., PDO, ENSO and NPGO) (Table 3). Our analyses demonstrate that POC is only negatively correlated with the NPGO index in the Bohai Sea, but positively correlated with the PDO index and negatively correlated with the NPGO index and SOI in the Yellow Sea. Apparently, large-scale physical processes (e.g., ocean circulation and ocean-atmosphere interaction) have significant impacts on the interannual variability of POC in the Yellow-Bohai Sea.

**Table 3:** The correlation coefficients between climate mode (PDO, NPGO and SOI) and POC in different areas.

	Bohai Sea	North Yellow Sea	South Yellow Sea	Total
PDO	0.091	0.166*	0.300**	0.259**
NPGO	−0.325**	−0.218**	−0.357**	−0.363**
SOI	−0.091	−0.260**	−0.195*	−0.192**

p > 0.05; *p < 0.05; **p < 0.01.

## Conclusions and Future Work

Our study shows large spatial and temporal variations in the surface POC of the Yellow-Bohai Sea. Surface POC is generally higher in the Bohai Sea (315–588 mg m^−3^) than in the Yellow Sea (181–492 mg m^−3^), with a declining trend from the nearshore to offshore. Mean POC is highest in spring for the whole Yellow-Bohai Sea and lowest in winter in the Bohai Sea but in summer in the Yellow Sea. The spatial and seasonal patterns of POC are attributable to complex influences of primary productivity, sediment resuspension, water exchange and terrestrial inputs. Our analyses show an overall decreasing trend in POC prior to year 2012 but upward trend until the end of 2015, which is almost opposite to chlorophyll. The decline (prior to 2012) might be associated with a decline in riverine inputs of allochthonous POC into the Yellow-Bohai Sea and a strengthened water exchange with the East China Sea. However, the increase during 2012–2015 (mainly in the fall-winter season) was a result of a decreased water exchange with the East China Sea that might be associated with the weakened Yellow Sea Warm Current. Preliminary analysis indicates that climate modes of ENSO, PDO and NPGO may have various impacts on the surface POC in the Yellow-Bohai Sea. Future studies with quantitative approaches are needed to investigate the different influences of biological productivity, sediment resuspension, physical transport and terrestrial inputs at various time scales to better understand the climate and anthropogenic impacts on the dynamics and fate of POC in the Yellow-Bohai Sea.

## Materials and Methods

### Data sources

The primary data **s**et is monthly mean values of surface POC derived from MODIS-Aqua (https://oceandata.sci.gsfc.nasa.gov). The satellite mission provides global coverage of remote sensed reflectances in selected spectral bands in the visible and near infrared spectral bands at approximately every 2 days^24^. We use the Level 3 POC data with a 4 km resolution (Standard Mapped Images, SMI) from July 2002 to December 2016, which was based on an algorithm by Stramski *et al*.^60^. This algorithm uses an empirical relationship derived from *in situ* measurements of POC and blue-to-green band ratios of spectral remotely sensed reflectances (R_rs_(λ)) to calculate the concentration of POC^60^, which is:

$${\rm{POC}}={\rm{a}}\times {(\frac{Rrs(443)}{Rrs(555)})}^{b}$$

where a and b is set as 203.2 and −1.034, respectively. We use Ferret software to fill the missing values with linear interpolation from the nearest surrounding points. In this study, we consider three sub-regions, i.e. the Bohai Sea (118.5°E:121°E, 37°N:40°N), the north Yellow Sea (121°E:124.5°E, 37°N:40°N) and the south Yellow Sea (119°E:126°E, 33°N:37°N).

We collect monthly data of three climate modes indices, i.e., PDO index (http://jisao.washington.edu/pdo/), NPGO index (http://www.o3d.org/npgo/) and Southern Oscillation Index (SOI) (http://www.cpc.ncep.noaa.gov/data/indices/soi) from January 2003 to December 2016. The PDO and NPGO indices of climate variability emerge from analyses of northeast Pacific sea-surface temperature anomalies and sea-surface height anomalies over the region (180°W–110°W; 25°N–62°N), which are the first and second corresponding principal components using empirical orthogonal function techniques^21^. The SOI is one measure of the large-scale fluctuations in air pressure occurring between the western and eastern tropical Pacific (i.e., the state of the Southern Oscillation) during El Niño and La Niña episodes. In general, this index has been calculated based on the differences in air pressure anomaly between Tahiti and Darwin, Australia. We carry out correlation analyses to assess the relationship between POC anomaly and climate modes through using SPSS software to calculate the correlation coefficient between monthly mean POC in different regions and climate modes.

### Data availability

The data are available at https://oceandata.sci.gsfc.nasa.gov.
